# Investigation of the Allelopathic Effect of Two Invasive Plant Species in Rhizotron System

**DOI:** 10.3390/life14040475

**Published:** 2024-04-04

**Authors:** László Bakacsy, Luca Viktória Kardos, Ágnes Szepesi, Krisztina Napsugár Nagy, Andrea Vasas, Gábor Feigl

**Affiliations:** 1 Department of Plant Biology, Institute of Biology, Faculty of Science and Informatics, University of Szeged, Közép fasor 52, 6726 Szeged, Hungary; kardosluca0330@gmail.hu (L.V.K.); szepesia@bio.u-szeged.hu (Á.S.); krisznapi@gmail.com (K.N.N.); 2 Doctoral School of Environmental Sciences, University of Szeged, Rerrich Béla tér 1, 6720 Szeged, Hungary; 3 Department of Pharmacognosy, Faculty of Pharmacy, University of Szeged, Eötvös u. 6, 6720 Szeged, Hungary; vasas.andrea@szte.hu

**Keywords:** allelopathy, invasive species, common milkweed, false indigo-bush, rhizotron

## Abstract

A key question in plant invasion biology is why invasive plants are more competitive in their introduced habitat than in their native habitat. Studies show that invasive species exhibit allelopathy, influencing other plants by releasing chemicals. Research on allelopathy uses *in vitro* tests, investigating effects on seed germination and seedling development. Although soil plays a role in modifying allelopathic effects, observations with soil are rare and almost nothing is known about the root development of test plants developing in soil and the effects of allelopathic compounds on root architecture. Our study evaluates the allelopathic effects of false indigo-bush (*Amorpha fruticosa* L.) and common milkweed (*Asclepias syriaca* L.) on oilseed rape growth as a model plant. The rhizotron system was used to study the effect of morphology and root architecture. Leaf–soil mixtures at 0.5%, 1%, and 5% concentrations were used. Shoot and root development was strongly inhibited at 5%. But there was no difference between the allelopathy of the two species, and the application of lower concentrations did not show any effect, demonstrating that soil has a significant modifying effect on their allelopathy. Our results highlight that the development of roots growing in the soil is also worth investigating in connection with allelopathy, which can strengthen the ecological importance of allelochemicals during successful invasions.

## 1. Introduction

Among alien species, invasive species can cause serious environmental problems by often outcompeting native flora and fauna through the colonization and alteration of their natural habitats. This makes biological invasions one of the most significant threats to the ecological balance and biodiversity of natural communities [1,2,3]. Due to their biological specificity, invasive species can cause significant economic damage and human health problems worldwide [4,5]. The total cost of the damage they cause and their management is comparable to that of natural disasters [6]. The defense against invasive species and their damage shows exponential growth, which a recent study predicts will cost a minimum of USD 20 billion annually in the European Union alone, starting from the 2020s [7].

A key question in plant invasion biology is why invasive plants are more competitive in their introduced habitat than in their native habitat [8,9]. Researchers have examined a number of traits to explain the increased competitiveness of invasive plant species, with one of the most prominent being the phenomenon of allelopathy [10]. In this process, plants influence the growth or development of another plant or other organism directly or indirectly by releasing chemicals into the environment [11]. The release of these chemicals into the environment can occur in a number of ways, e.g., in plants by volatilization from leaves, the leaching and decomposition of plant parts (leaves, stems, etc.), and root exudation [11,12]. This concept can encompass both positive and negative effects and, therefore, they are gaining increasing attention in agriculture as potential natural herbicides [13,14] in natural ecosystems; these types of plant interactions are of fundamental importance [15,16]. Allelopathic compounds may also play a protective role in a number of invasive plants [17,18]. Some invasive plants use allelochemicals to inhibit soil-borne pathogens [17,19,20] or to deter insect pests [21,22], sometimes nematodes [23,24]. In addition to plant–plant interactions, allelopathy can also mediate competitive interactions between plant species [17,18]. Experiments show that chemicals released by some invasive plant species are more effective against native plant species in new areas they invade. This is not surprising, as the so-called “novel weapon” hypothesis suggests that during evolution, co-evolving species living in close proximity to each other have successfully adapted to each other’s metabolites, whereas an alien species “from afar” may be more likely to produce compounds to which native plants are less resistant [25,26]. For example, (±)-catechin [27] released by *Centaurea maculosa* Lam. and 7,8-benzoflavone [28] released by *Acroptilon repens* L. have had allelopathic effects on native plants in invaded areas. It has also been shown in several cases that invasive species not only maintain but even increase the production of allelopathic compounds in their new range if they provide a competitive advantage over native plants [29,30].

Typically, allelopathy is detected by *in vitro* tests, where the most common method is to study the effect of aqueous solutions of a given plant or plant part on seed germination and seedling development in test plants, usually in a simple system (e.g., Petri dish, filter paper). Of course, these *in vitro* methods are essential for detecting allelopathy. However, laboratory studies analyzing the functions of either the extracts or the isolated chemicals cannot detect the significance of allelopathy in communities [31]. In addition, this type of study may significantly overestimate the effects of the plants and their materials under study [32], because under natural conditions, allelopathic compounds are transported into a complex plant–soil system where they can only reach their target by crossing many barriers, and their effects can vary considerably, usually being attenuated, but sometimes persisting or even being enhanced [33,34]. One of these modifying factors may be leaching by precipitation, which dilutes the active substance and washes it into the deeper soil layers, so the manifestation of allelopathy is often more pronounced in arid or semi-arid environments [35]. Another factor is the binding of allelopathic material to soil particles and its reaction with microorganisms. Research by Blum [36] and Blum et al. [37,38] showed that in sterile soil, 29% of the introduced compound (ferulic acid) was irreversibly bound to soil particles, 8% was reversibly bound, while 63% was in soil solution. Reversible sequestration plays an important role in preventing microbial degradation of the active compound, making the effect more likely to manifest. According to Dalton [39], the reversible binding of allelopathic substances to soil particles can prevent their microbial degradation, allowing their effect to be exerted. Irreversibly bound phenolics are also important for compound availability and can be taken up by fungal hyphae [40]. This can be exploited to reduce or even prevent their negative effects by binding them to the soil (or by degradation by microbes). In several cases, the addition of activated carbon to soil has been shown to reduce the effects of allelopathic substances, allowing native species to thrive while invasive species fare worse [41,42,43]. For example, Vivanco et al. [25] found that the removal of allelopathic material from North American soils of the invasive *Centaurea diffusa* Lam. was more effective in inhibiting the growth of the invasive species than in the soil of the invasive species’ native range (Eurasia). This suggests that the soil biota in the new habitats of the invasive species is not accustomed to the allelochemicals of the invasive species and may therefore also promote invasion. Contrary results are known; e.g., Ito et al. [44] investigated the active compound of the invasive *Solidago altissima* L. in soil. They found that the compound was no longer detectable after ten days, suggesting that microorganisms play an important role in degrading the compound. Although the allelopathic substances are bound and degraded by microbes, the test plant often grows poorly because the microorganisms extract nitrogen from the plants and become their competitors [45].

Several studies have therefore shown that it would be useful to test the allelopathic potential of an invasive species under field conditions [31,38,46]. The allelopathy test conducted in the field, however, is extremely difficult to carry out, if it possible [31,38]. Therefore, only allelopathic potential can be tested under artificial laboratory conditions, but this can provide important information. Research on allelopathy has predominantly focused on allelochemicals and *in vitro* germination assays, so, for example, soil-based treatments and/or longer time course studies are less commonly used. In addition, little is known about the effects on root development, architecture, and morphology [47], even though such compounds affect the development of neighboring plants through the root in addition to germination [16,47] and thus also affect above-ground parts (shoots) [14]. Therefore, by studying allelopathy in a plant–soil or root–soil system, we can now move closer to more complex systems that better model the field environment and we can thus better understand the role of allelopathy in natural communities.

The use of soil-filled rhizotrons can be a good compromise between *in vitro* systems and field studies, allowing real-time, non-invasive studies of root system development [48]. Root responses of agricultural crops to different stressors have been investigated in such systems in several cases, e.g., treated with zinc [49] or nickel [50], combined heavy metal treatment modeling wastewater [51], polypropylene surgical mask fragments [52], or raw and nutrient-enriched pomegranate peel powder [53].

The allelopathic effects of the two most common invasive plant species in the EU, the false indigo-bush (*Amorpha fruticosa* L.) and the common milkweed (*Asclepias syriaca* L.), are well known from the literature [46,54], but we do not know how these species inhibit the development of other plants in the soil environment, particularly with regard to their root architecture. The shrub false indigo-bush (*A. fruticosa*) is an aggressive, common species of scrub and floodplain and thrives on a wide range of soil types. It begins to leaf in May and sheds its leaves in October. From a conservation point of view, it is a threat to native floodplain vegetation, especially grassland, and forms monodominant stands by spreading rapidly and growing in masses. It reduces the functioning and plant diversity of the ecosystems and invades by altering carbon and nitrogen cycles (N_2_ fixation) [55]. It is a difficult weed to control in floodplain afforestation, and arable land and grassland management. Due to its allelopathic substances, its rapid invasion of agricultural fields can negatively affect the growth and yield of valuable forage and agricultural crops [56]. It is also a serious problem from the perspective of flood control [57]. The herbaceous invasive species common milkweed (*A. syriaca*) occurs in Central Europe on less compacted, sandy soils. There is detailed knowledge of its effects on natural vegetation, but it also causes significant economic damage [58,59]. According to the Fifth National Weed Survey, about 16,000 hectares of arable land was infested, but it mainly weeds planted poplar, sand pine plantations, vineyards, and orchards [60]. Yenish et al. [61] and Varga and Dancza [60] reported yield losses in wheat. Its allelopathic effect may play an important role in the damage of both species [46,54,62].

The aim of our study was to determine whether the effect of allelopathy is manifested when leaf litter from these two invasive plant species is mixed into soil. We also wanted to determine whether the two invasive plant species, when added to soil, had any effect on the root or shoot development of the test plants and, if so, how and at what concentrations the effect can be observed. To find out, the shoot development and root architecture of the test plant oilseed rape (*Brassica napus* L.) were examined after two weeks of growth in rhizotrons.

## 2. Materials and Methods

### 2.1. Application of Plant Material and Model Plant

To investigate the expression of allelopathy in the soil, we mixed ground leaves of two invasive plant species at different concentrations into the soil. While the use of ground leaves as an extraction method may be considered crude, it allowed for the collection of representative mixtures of allelochemicals present in invasive species leaves, enabling us to assess their overall allelopathic impact on test plant growth in a soil-based system. The utilization of ground leaf material offers distinct advantages over isolated active ingredients, as it encompasses a complex mixture of compounds present in the plant tissue, thereby better simulating the natural decomposition process and allelopathic interactions observed in ecological settings (c.f. Pardo-Muras et al. and Weidenhamer et al. [63,64]). For this purpose, we collected healthy and undamaged mature leaves of the adventive false indigo-bush (*A. fruticosa*) and common milkweed (*A. syriaca*) from the floodplain of the lower section of the Tisza River near Szeged, Hungary, in August 2022. The collection was carried out in non-agricultural areas to avoid potential misleading effects from possible herbicide treatments. After collection, the leaves were dried in darkness at room temperature and ground into a powder using a Retsch SM 100 comfort grinder (Department of Pharmacognosy, University of Szeged, Hungary) with a mesh size of 2 mm. As a test plant, oilseed rape (*Brassica napus* L. cv. GK Gabriella) provided by the Cereal Research Non-profit Ltd., Szeged, Hungary, was used. Oilseed rape has significant economic and agricultural importance worldwide. It is sensitive to abiotic and biotic stresses, which have been the focus of numerous studies, and it is also frequently used as a model plant in allelopathic research.

### 2.2. Soil Properties, Growth Conditions, and Rhizotron System 

For the allelopathic investigation, following the experimental setup of Mészáros et al. [52], rhizotrons were used that were 15 cm wide, 30 cm tall, and 1.6 cm thick (with a filling strip thickness of 1 cm). FLORIMO general-purpose potting soil (Matécsa Gardening Ltd., Kecel, Hungary) was used for this study. Its pH value was 6.4 ± 0.5, its organic matter content was 70% (*w*/*w*), K_2_O was 0.3% (*w*/*w*) (min. 3000 mg/kg), P_2_O_5_ was 0.1% (*w*/*w*) (min. 1000 mg/kg), and N was 1.0% (*w*/*w*) (min. 10,000 mg/kg). The soil was stored at −20 °C in a refrigerator for 2 weeks for sterilization purposes. To achieve the appropriate soil structure, 20% sand was mixed with the potting soil. For the treatments, three concentrations were applied by adding plant material to the soil mixture at ratios of 0.5%, 1%, and 5% (*w*/*w*). The plant material was manually mixed to homogenize it. In our experiment, considering 1500 g of soil mixture, 7.5 g (0.5%), 15 g (1%), and 75 g (5%) of plant material were added. The percentage concentrations correspond to those used by Csiszár et al. [46], but it is important to note that in their case, the plant material was soaked in water (they used aqueous extract). An average of 300 g of soil mixture was filled into each rhizotron the day before planting, and the soil mixtures had an average initial moisture content of 70% [52]. Before planting, the *B. napus* seeds were pre-germinated in darkness at 26 °C for 24 h. Then, one pre-germinated seed was placed on the surface of each pre-filled rhizotron. The rhizotrons containing the germinated seeds were covered with transparent plastic foil for the first 48 h to maintain optimal humidity. The growth period lasted for a total of 14 days, during which the plants were watered with 10 mL of distilled water every other day. The plants were grown under greenhouse conditions (Figure S1) with a lighting cycle of 12/12 h day/night, irradiance of 41.1 W m^−2^, photon flux density of 199.6 µmol m^−2^ s^−1^, and 9680 lux, determined using a SpectraPen MINI (PSI, Drásov, Czech Republic) instrument. The temperature was maintained at 24/22 °C, and the relative humidity was 55–60%.

### 2.3. Morphological Measurements 

**Figure 1:**
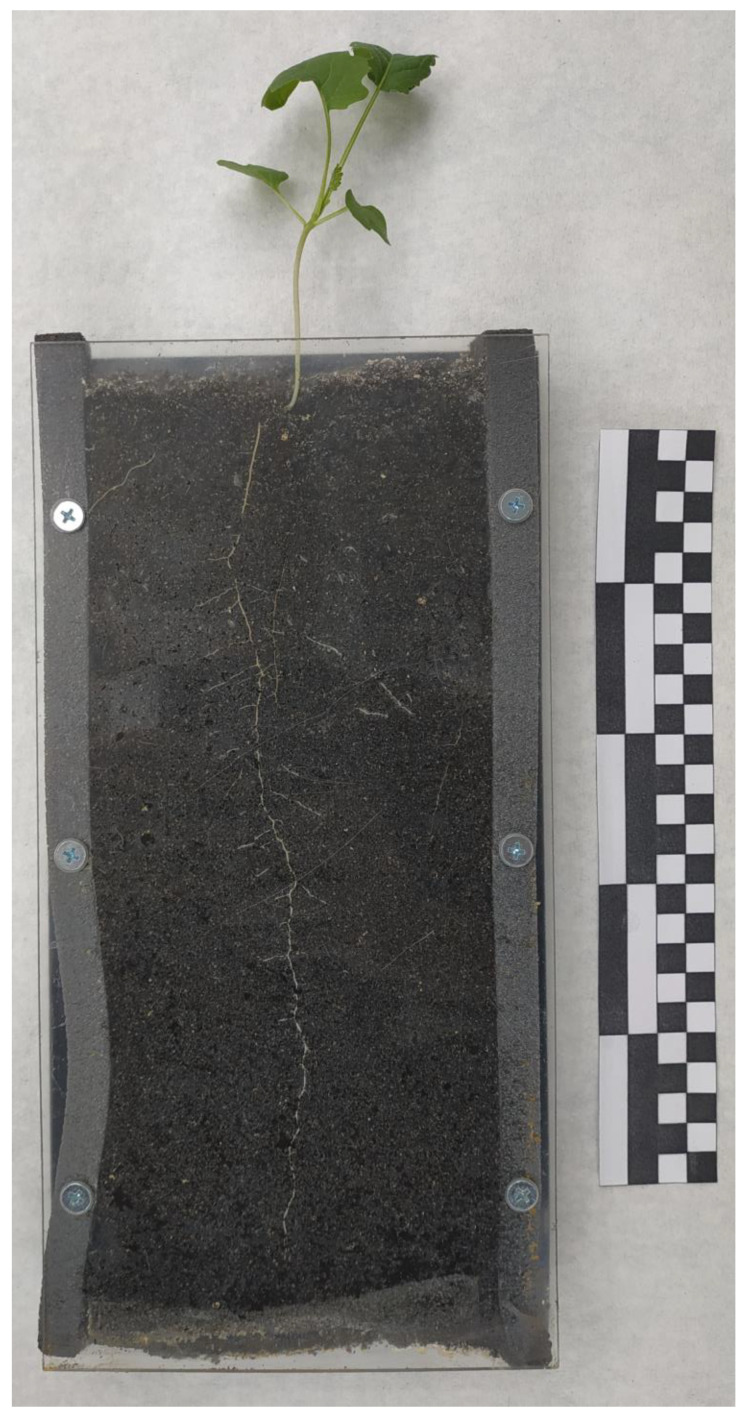
A 14-day control rapeseed was grown in rhizotron. In the image, the primary root and lateral roots of the plant are clearly visible, growing along the walls of the rhizotron in the soil.

At the end of the 14-day growth period, the plants were scanned using Czur Shine 800 Pro scanner (Czur Tech Co., Ltd., Dalian, China), and their morphological characteristics were evaluated using Fiji (ImageJ) software, version number 1.54a [65]. The following parameters were measured for the plants: shoot length (measured from the root collar to the last leaf node in centimeters), number of leaves, and leaf area (cm^2^) (Figure S2). Additionally, the root architecture of the plants was assessed, including the length of the primary root (cm), number of lateral roots, and calculation of lateral root density (number of lateral roots per unit primary root length) (Figure 1).

### 2.4. Statistical Analysis

The presented data represent the mean values of three independent experiments. Three replicates were conducted for both the false indigo-bush and common milkweed, with each replicate including five parallels to test the expression of allelopathic effects on shoot and root morphology in the soil. Statistical analysis was performed using GraphPad Prism for Windows version 8.0.1.244 (GraphPad Software, La Jolla, CA, USA). Normality testing was conducted using the Shapiro–Wilk test. To determine statistically significant differences, one-way ANOVA was used for datasets that followed a normal distribution, while the Kruskal–Wallis test was used for datasets that did not follow a normal distribution. Results were considered significant at *p* ≤ 0.05.

## 3. Results

### 3.1. Results of Shoot Morphological Measurements 

**Figure 2:**
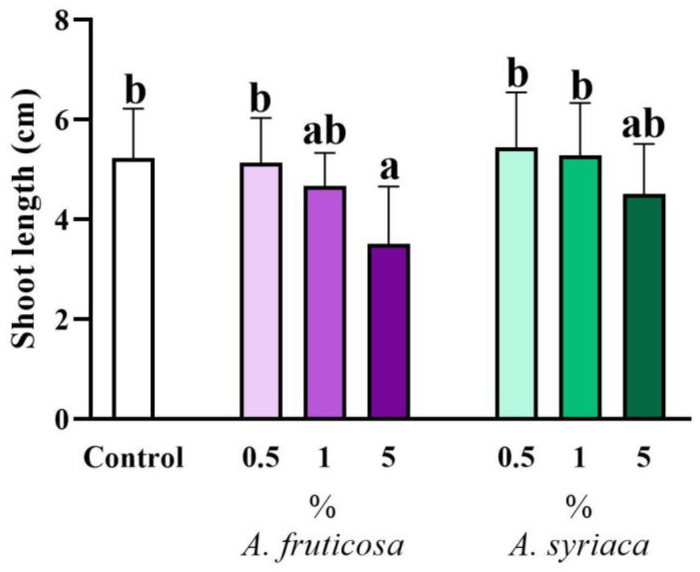
The allelopathic effect of leaf extracts at different concentrations of the two invasive plant species on the shoot length of *Brassica napus* after 14 days in a plant–soil system. Different letters indicate significant differences according to one-way ANOVA (*p* ≤ 0.05, data presented as mean ± SD, *n* = 15). “a” represents the most prominent group, “b” indicates milder differences. Columns marked with the same letter show no significant difference (the values of the significant differences can be found in Table S1).

After 14 days of cultivation, the shoot length of rapeseed did not show any significant difference compared to the control when ground leaves of the invasive species were applied at the two lower concentrations (0.5% and 1%). However, among the ground leaves mixed with soil at a high concentration (5%), only the presence of false indigo-bush showed a strong inhibitory effect on shoot elongation, while milkweed did not cause any difference at any concentration compared to the control. There was no significant difference between treatments with the same invasive species at the lower concentrations (0.5% and 1%) (Figure 2). However, there was a significant difference between treatments with 0.5% and 5% ground false indigo-bush leaves. For milkweed, there was no significant difference between the lower concentrations and the 5% treatment. When comparing the effects of ground leaves from the two invasive species, significant differences were observed only between the higher concentration of false indigo-bush and the lower concentrations of milkweed: there was a significant difference between the 5% treatment of false indigo-bush and the 0.5% and 1% treatments of milkweed. There was no statistically significant difference between ground leaves of the two invasive species applied at the high concentration (Figure 2).

**Figure 3:**
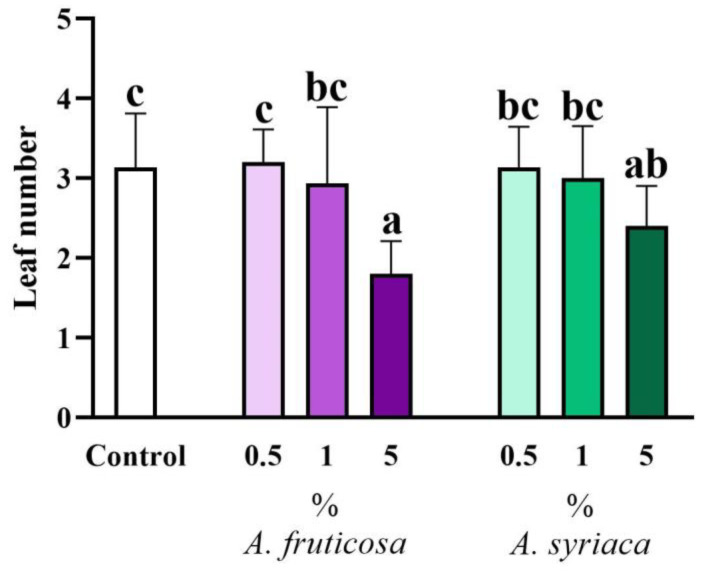
The allelopathic effect of leaf extracts at different concentrations of the two invasive plant species on the leaf number of *Brassica napus* after 14 days in a plant–soil system. Different letters indicate significant differences according to one-way ANOVA (*p* ≤ 0.05, data presented as mean ± SD, *n* = 15). “a” represents the most prominent group, “b” indicates milder differences, while “c” signifies another group. Columns marked with the same letter show no significant difference (the values of the significant differences can be found in Table S1).

The effect of the ground leaves of the two species mixed into the soil on the leaf numbers of rapeseed is presented in Figure 3. The inhibitory allelopathic effect of the two invasive species on leaf numbers was only significant at the 5% concentrations compared to the control: the 5% treatment with false indigo-bush resulted in significantly fewer leaves compared to the control, while the same concentration of milkweed had a similar but weaker effect, which was still significant (Figure 3). There was also a significant difference in leaf numbers between the 5% treatment of false indigo-bush and its 0.5% and 1% treatments. Similar to the shoot lengths, it is worth noting that there was no difference in inhibitory effects on leaf numbers between the high concentrations of the two invasive species (Figure 3). However, there were differences between the 5% treatment of false indigo-bush and the 0.5% and 1% treatments of milkweed, as well as between the 5% treatment of milkweed and the 0.5% treatment of false indigo-bush in terms of their effects on leaf numbers (Figure 3).

**Figure 4:**
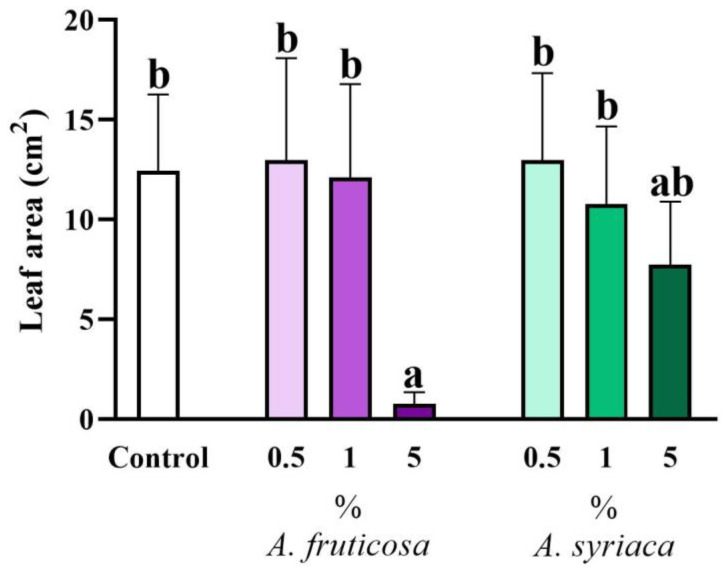
The allelopathic effect of leaf extracts at different concentrations of the two invasive plant species on the leaf area of *Brassica napus* after 14 days in a plant–soil system. Different letters indicate significant differences according to one-way ANOVA (*p* ≤ 0.05, data presented as mean ± SD, *n* = 15). “a” represents the most prominent group, “b” indicates milder differences. Columns marked with the same letter show no significant difference (the values of the significant differences can be found in Table S1).

Interestingly, when examining the effect on leaf area, only the highest concentration of false indigo-bush showed a very strong inhibitory effect compared to the control and other treatments. In contrast, it was inhibited only in the 5% milkweed treatment, albeit slightly, and no inhibitory effect was detected in the other treatments (Figure 4). However, there was no significant difference in the effect on leaf area between treatments with the two species, despite noticeable visual differences (Figure 4).

### 3.2. Results of Root Morphological Measurements 

**Figure 5:**
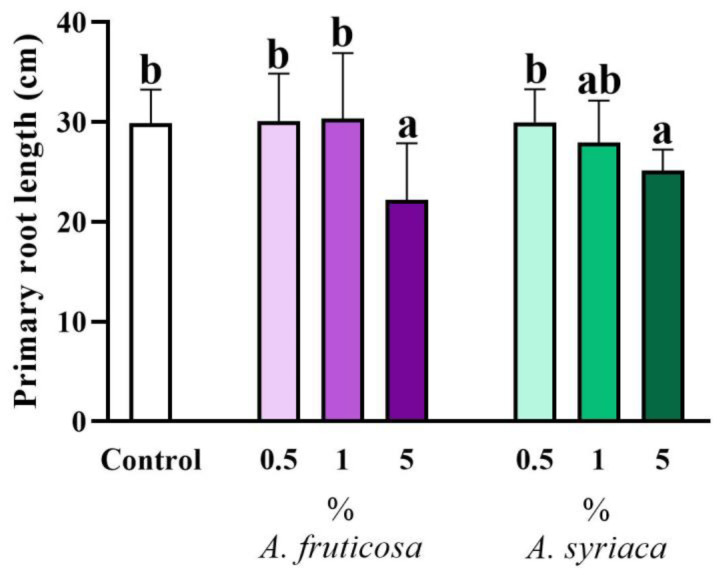
The allelopathic effect of leaf extracts at different concentrations of the two invasive plant species on the primary root length of *Brassica napus* after 14 days in a plant–soil system. Different letters indicate significant differences according to one-way ANOVA (*p* ≤ 0.05, data presented as mean ± SD, *n* = 15). “a” represents the most prominent group, “b” indicates milder differences. Columns marked with the same letter show no significant difference (the values of the significant differences can be found in Table S2).

The 14-day cultivation in rhizotrons allowed for the investigation of the allelopathic effects on the root system and its non-invasive measurement (Figure S1). Among these measurements, we first observed the effect of allelopathy on the primary root length, as shown in Figure 5. Similar to the above-ground morphological features, no significant differences were found in the case of 0.5 and 1% applications of ground leaves of invasive species in terms of primary root length, compared to the control. However, a significant inhibition of primary root elongation was observed in the 5% treatments compared to both the control and the lower concentrations. The 5% treatment with false indigo-bush exerted a significant inhibitory effect on the primary root length compared to the control and the other treatments. The 5% treatment with milkweed significantly inhibited primary root growth compared to the control and the 0.5% treatment, while it did not significantly inhibit growth compared to the 1% treatment (Figure 5). While there were no significant differences between treatments of the two species at the same concentration, significant differences were observed among different concentrations. Significant differences were also observed between the 5% treatment of false indigo-bush and the 0.5% treatment of milkweed. Moreover, there were significant differences between the 5% treatment of milkweed and the 0.5% and 1% treatments of false indigo-bush as well (Figure 5).

**Figure 6:**
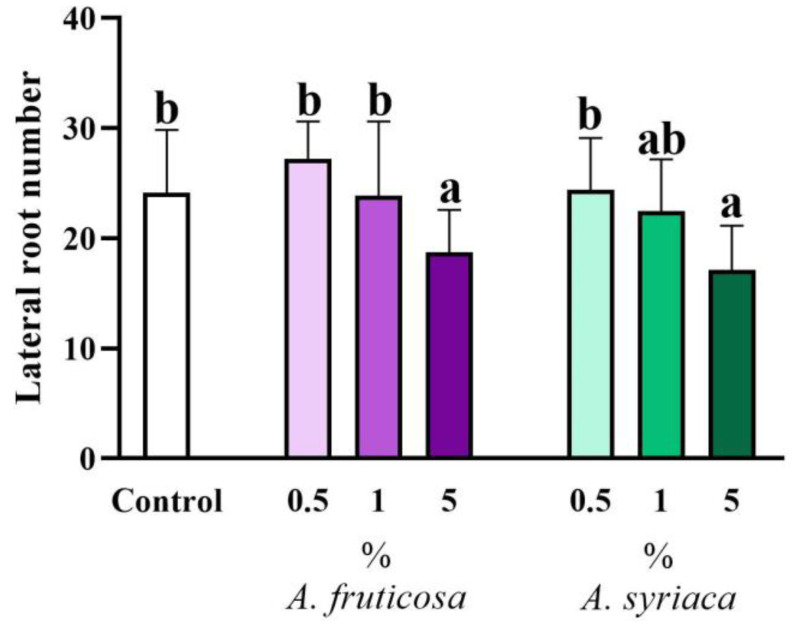
The allelopathic effect of leaf extracts at different concentrations of the two invasive plant species on the number of lateral roots of *Brassica napus* after 14 days in a plant–soil system. Different letters indicate significant differences according to one-way ANOVA (*p* ≤ 0.05, data presented as mean ± SD, *n* = 15). “a” represents the most prominent group, “b” indicates milder differences. Columns marked with the same letter show no significant difference (the values of the significant differences can be found in Table S2).

Similar changes can be observed in the case of the allelopathic effect on lateral roots as in the case of primary root lengths (Figure 6). Only the high concentrations of the two species caused a decrease in lateral root numbers compared to the control (Figure 6). Furthermore, the application of 5% false indigo-bush showed a significant difference compared to the 0.5% concentration of false indigo-bush and the 0.5% concentration of milkweed. The effect of the 5% ground leaf treatments of milkweed significantly differed from the 0.5% and 1% concentrations of false indigo-bush as well as the 0.5% concentration of milkweed (Figure 6). However, no significant difference was observed in the effect of the 5% concentrations of the two species; their effects on lateral root numbers were similar (Figure 6).

**Figure 7:**
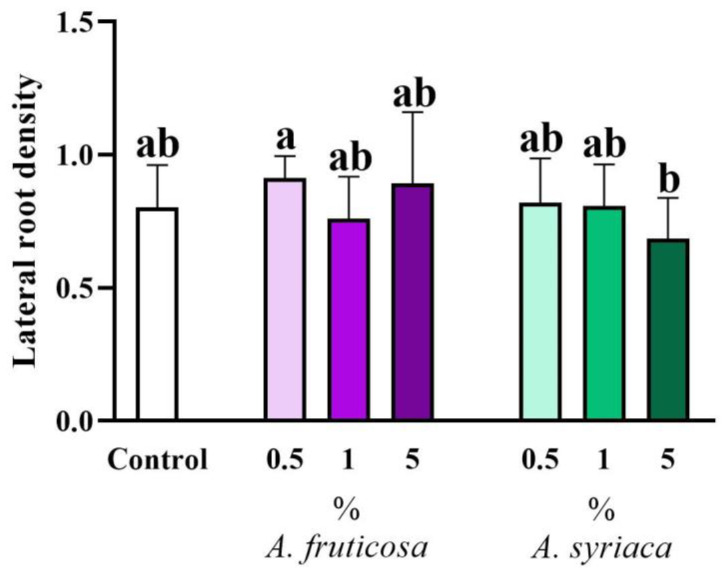
The allelopathic effect of different concentrations of leaf extracts from the two invasive plant species on the lateral root density of 14-day-old *Brassica napus* plants in a plant–soil system was investigated. Different letters indicate significant differences according to one-way ANOVA (*p* ≤ 0.05, data presented as mean ± SD, *n* = 15). “a” represents the most prominent group, “b” indicates milder differences. Columns marked with the same letter show no significant difference (the values of the significant differences can be found in Table S2).

In the case of non-lethal stress events, a commonly observed phenomenon is the development of a stress-induced morphogenic response (SIMR), characterized by the inhibition of primary root elongation and an increase in lateral root number [66], which likely enhances stress tolerance. In the present study, the root growth responses do not fully conform to the classical requirements of SIMR. However, it is worth noting that the treatment with false indigo-bush at a concentration of 0.5% resulted in increased lateral root density (Figure 7), suggesting a mechanism related to tolerance, as, in this case, the primary root length was not reduced (Figure 5). Conversely, treatments with milkweed exhibited a concentration-dependent reduction in lateral root density (Figure 7), which can be explained by the concurrent decrease in primary root length and lateral root number (Figure 5 and Figure 6).

## 4. Discussion

In our study, we tested the allelopathic effects of two invasive plant species on *B. napus* growth in a rhizotron system. We examined the impact of allelopathy on the shoot morphology of the test plant (shoot length; number and area of leaves), and primarily focused on the root architecture (main root length and number of lateral roots) in a soil-based system. Therefore, our study significantly advances the understanding of allelopathic interactions by directly comparing the species-specific effects of *A. fruticosa* and *A syriaca* on *B. napus* growth, providing novel insights into the allelopathic potential of invasive species in agroecosystems and invaded nature communities, respectively.

The application of the two species that are invasive in lower concentrations did not cause a statistically significant growth response compared to the control, and there was no significant difference in their effects on rapeseed growth. This differs from the results of previous *in vitro* tests reported in the literature. In the case of false indigo-bush, its 5% concentration showed a strong inhibitory effect on rapeseed shoot length, leaf number (Figure 2 and Figure 3), and main root length (Figure 5). These findings are consistent with the studies conducted by Csiszár et al. [32,46], which demonstrated that a 5% concentration aqueous extract of the species strongly inhibited shoot and root length in the test plant. In contrast to our results, their study conducted on 6-day-old white mustard (*Sinapis alba* L.) as a model plant showed a similarly strong inhibitory effect even at the 1% treatment, and they also demonstrated that the effect of *A. fruticosa* was among the strongest inhibitory effects observed among the studied invasive species, at all concentrations (including compared to milkweed). The difference can presumably be explained by the modifying effect of the soil, which in our case weakened the allelopathic inhibitory effect at lower concentrations and made it similar to milkweed. However, we did not observe the growth of the test plants, which would suggest that the test plant could utilize the ground material mixed into the soil as a nutrient. When applied in higher concentrations, the ground materials clearly exhibit their negative allelopathic effect. This is supported by the study of Bodor et al. [53], in which they demonstrated that powder made from pomegranate peel had a beneficial effect on the model plant when applied in the soil, resulting in the growth of its above-ground parts. This theory is supported by the results of Kazinczi et al. [67], who investigated the allelopathy of several invasive plant species in potted experiments under greenhouse conditions and found stimulating effects in some species, which facilitated the development of cultivated plants, which indicates, on the one hand, the buffering capacity of the soil, and, on the other hand, that each species has a different allelopathic effect. Nagy et al. [68], for instance, demonstrated that the allelopathy of the Indian blanket flower (*Gaillardia pulchella* Foug.) exerts distinct effects on the development of the test plants. The aqueous extract of the blanket flower caused a significant elongation of shoots in *B. napus* seedlings, while inhibiting the growth of their roots. Novak et al. [69] also examined the allelopathic effect of aqueous extracts of several invasive species, including false indigo-bush, on rapeseed (*Brassica napus*), sunflower (*Helianthus annuus* L.), and oat (*Avena sativa* L.) test plants in Petri dishes during a 4-day growth period. Similar to our findings, the germination and growth of the seedlings were significantly inhibited by the aqueous extract of false indigo-bush, with the strongest negative effect observed on shoot growth. Krstin et al. [56] also characterized the phytotoxic effects of aqueous leaf extracts obtained from *A. fruticosa* (1%, 3%, and 5% concentrations) in an *in vitro* study on the germination and growth of four important agricultural species (*Helianthus annuus* L., *Medicago sativa* L., *Trifolium pratense* L., and *Triticum aestivum* L.). Similar to our findings, their results showed that the phytotoxicity of false indigo-bush leaf extract depended on its concentration, with higher concentrations exhibiting stronger phytotoxic effects. The highest concentration (5%) of leaf extract caused inhibition similar to juglone in the germination and growth of the test plants. In the present study, it was also observed that the lowest-concentration (0.5%) treatment with false indigo-bush significantly increased the number of lateral roots in rapeseed compared to the control and other treatments (Figure 6).

The effect of ground leaves of milkweed in 5% in soil also caused significant inhibition in the measured parameters, which was statistically equivalent to the effect of false indigo-bush. The application of milkweed residue had a nearly identical effect on rapeseed shoot length, leaf count, and side root count as false indigo-bush (Figure 2, Figure 3 and Figure 6). This is somewhat supported by the work of Csiszár et al. [46], in which milkweed aqueous extract was applied at two concentrations (1% and 5%): the higher concentration of the extract had inhibitory effects on the shoot length of 6-day-old white mustard, while both concentrations had strong, significantly negative effects on root length compared to the control plants. The allelopathic effect of milkweed was inferior to that of false indigo-bush, as can be perceived from the impact on leaf area of test plants in this study, although not to a significant extent (Figure 4). Popov et al. [70] also tested the allelopathic potential of milkweed by examining its root aqueous and methanol extracts on three agricultural crops: corn (*Zea mays* L.), soybean (*Glycine max* (L.) Merr.), and sunflower (*Helianthus annuus* L.). They observed a significant inhibition of germination in all three plant species even at the lowest concentration of the aqueous extract, while no inhibition was observed compared to the control with the methanol extract. Additionally, both types of extracts significantly reduced shoot and root length in all tested plants, which is consistent with our study on 14-day-old field-grown rapeseed (Figure 2 and Figure 5). Several other publications also demonstrate that the milkweed aqueous extract inhibited the germination and growth of test plants under laboratory conditions [71,72]. These findings, along with our results, somewhat contradict the study by Kazinczi et al. [67], who reported a stimulatory effect on the growth of corn and sunflower when they applied *A. syriaca* root residues in potted soil experiments. This, again, points to the modifying effect of the soil on the one hand, and the diverse allelopathic effects of the species on the other.

It is worth noting that some authors draw attention to the species-specific inhibition of false indigo-bush through allelopathy [56,69], while in contrast, we did not find any mention of this in the literature for common milkweed. The work of Krstin et al. [56] shows that the extracts of *A. fruticosa* reduced the germination of alfalfa and clover in a concentration-dependent manner, which agrees with our results, namely that the shoot length, leaf area, and number of lateral roots of *B. napus* decreased in a concentration-dependent manner (Figure 2, Figure 3 and Figure 6). However, in their work, they did not show any effect on sunflower, and in the case of wheat, it reduced its germination in a non-concentration-dependent manner. In the case of sunflower, Novák et al. [69] found that the leaf extract of false indigo-bush had a weak but significant inhibitory effect on its radicle and shoot length, while this effect was stronger for oats and rapeseed. The extract of the species mainly reduced the growth of radicle lengths in *Avena sativa*. In the case of *A. syriaca* treatment, a concentration dependence was found for the inhibition of the leaf area, primary root length, and number and density of lateral roots (Figure 4, Figure 5, Figure 6 and Figure 7). This is also confirmed by the studies of Popov et al. [70] and Szilágyi et al. [72], but interestingly, no attention was paid to this. Also, Popov et al. [70] showed that milkweed extract had a stronger negative effect on sunflower than on soybean and maize.

In summary, the growth of test plants was significantly inhibited by the ground leaves of both invasive species when applied at a concentration of 5% mixed with the soil. This finding is partly supported by previous *in vitro* studies conducted with the two invasive species, although those often showed strong inhibitory effects even at lower concentrations. This discrepancy may be due to the fact that under *in vitro* conditions, the test plants grown on aqueous solutions can come into contact with the allelopathic substance over a larger surface area even at lower concentrations, which could explain the seemingly stronger effect at lower concentrations. In our study, when using the allelopathic ground leaves at lower concentrations, contrary to the literature, it resulted in control-like shoot and root development, which can be attributed to the modifying effect of the soil. In our system, simulating more natural conditions, only the roots of the test plants are exposed to the allelopathic substance, while the shoots are positioned aboveground, not being in contact with the substances. We extended our investigations in the soil to assess the effect of allelopathy on root architecture, which, in both species, inhibited primary root elongation and lateral root formation at high concentrations (Figure 5 and Figure 6). This observation is supported by Bais et al. [47], who treated *Arabidopsis thaliana* (L.) Heynh. with the allelochemical compound ((-)-catechin) from *Centaurea maculosa* Lam. under laboratory conditions and demonstrated the induction of reactive oxygen species (ROS) waves in the root meristem, leading to gene expression changes and ultimately resulting in root system decay. Furthermore, they also confirmed under field conditions that the invasive species outcompetes native plant species by releasing the allelochemical substance from their roots.

## 5. Conclusions

This study investigates the ecological role of plant allelochemicals in the success of invasive species, specifically *Amorpha fruticosa* and *Asclepias syriaca*. Using oilseed rape as a test plant, this study examines the soil manifestation of allelopathy over 14 days. The focus is on how allelopathy affects the development of the test plants both above and below ground. The plants were grown in a rhizotron system to analyze shoot and root morphology. This study concludes that despite soil modifying the effects of allelochemicals, they still significantly impact root development. This research aids in understanding the success of certain invasive species. Future studies could complement our findings by conducting detailed chemical analyses to elucidate the specific fractions and structures of these allelochemicals.

## Data Availability

All data are contained within the article or Supplementary Materials.

## Supplementary Materials

The following supporting information can be downloaded at https://www.mdpi.com/article/10.3390/life14040475/s1. Figure S1: The growing conditions in greenhouse; Figure S2: Scanned leaves for measurement; Table S1: The values of the significant differences of shoot parameters; Table S2: The values of the significant differences of root parameters.
