# Supplementary material for: Investigation of the Allelopathic Effect of Two Invasive Plant Species in Rhizotron System
Source: Life (Basel). 2024 Apr 4;14(4):475. doi: 10.3390/life14040475 (PMC11051222; doi:10.3390/life14040475)
Supplement: Supplementary file 1 [file life-14-00475-s001.zip › life-2924675-supplementary.pdf]

Investigation of the Allelopathic Effect of Two Invasive Plant Species in Rhizotron System

László Bakacsy [1,*], Luca Viktória Kardos [1], Ágnes Szepesi [1], Krisztina Napsugár Nagy [1,2], Andrea Vasas [3] and Gábor Feigl [1,*]

[1] Department of Plant Biology, Institute of Biology, Faculty of Science and Informatics, University of Szeged, Közép fasor 52, 6726 Szeged, Hungary; kardosluca0330@gmail.hu (L.V.K.); szepesia@bio.u-szeged.hu (Á.S.); krisznapi@gmail.com (K.N.N.)
[2] Doctoral School of Environmental Sciences, University of Szeged, Rerrich Béla tér 1, 6720 Szeged, Hungary
[3] Department of Pharmacognosy, Faculty of Pharmacy, University of Szeged, Eötvös u. 6, 6720 Szeged, Hungary; vasas.andrea@szte.hu
* Correspondence: bakacsy@bio.u-szeged.hu (L.B.); feigl@bio.u-szeged.hu (G.F.)

Supplementary Materials

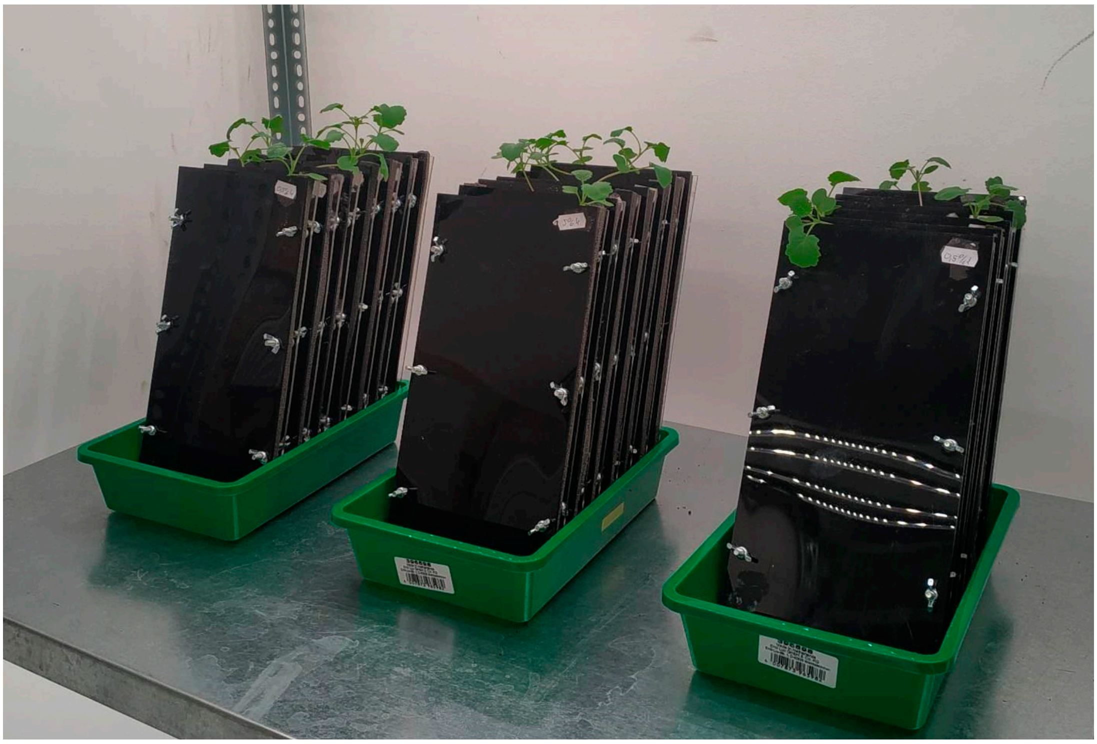

Figure S1. Growing conditions for 14 days in the rhizotrons. The rhizotrons were placed at an approximate tilt of 120°-130° to allow the roots of the test plants to grow along the transparent walls, ensuring their visibility and measurability.

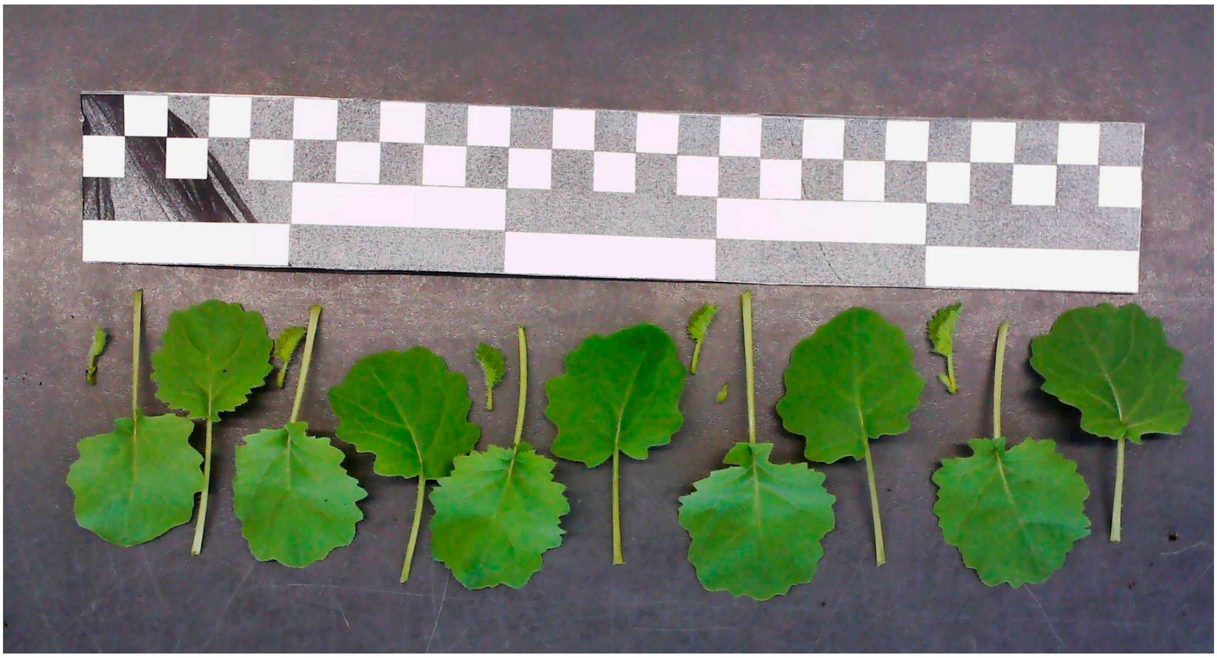

Figure S2. To measure the number and area of the leaves, they were scanned (the leaves of the control plants are visible in the image).

Table S1. The values of the significant differences of shoot parameters.

**Shoot length**	***p* value**
Control vs. False indigo-bush 5%	0.0003
False indigo-bush 0.5% vs. False indigo-bush 5%	0.0226
False indigo-bush 5% vs. Common milkweed 0.5%	0.0008
False indigo-bush 5% vs. Common milkweed 1%	0.0053
**Leaf number**	
Control vs. False indigo-bush 5%	< 0.0001
Control vs. Common milkweed 5%	0.0179
False indigo-bush 0.5% vs. False indigo 5%	< 0.0001
False indigo-bush 1% vs. False indigo 5%	0.0002
False indigo-bush 5% vs. Common milkweed 0.5%	< 0.0001
False indigo-bush 5% vs. Common milkweed 1%	0.0003
False indigo-bush 0.5% vs. Common milkweed 5%	0.0283
**Leaf area**	
False indigo-bush 5% vs. All treatments	< 0.0001

Table S2. The values of the significant differences of root parameters.

**Primary root length**	***p* value**
Control vs. False indigo-bush 5%	0.0006
Control vs. Common milkweed 5%	0.006
False indigo-bush 0.5% vs. False indigo-bush 5%	0.0009
False indigo-bush 1% vs. False indigo-bush 5%	< 0.0001
False indigo-bush 5% vs. Common milkweed 0.5%	0.0072
False indigo-bush 0.5% vs. Common milkweed 5%	0.006
False indigo-bush 1% vs. Common milkweed 5%	0.0005
Common milkweed 5% vs. Common milkweed 0.5%	0.0418
**Lateral root number**	
Control vs. False indigo-bush 5%	0.012
Control vs. Common milkweed 5%	0.0012
False indigo-bush 5% vs. False indigo-bush 0.5%	< 0.0001
False indigo-bush 5% vs. Common milkweed 0.5%	0.0282
False indigo-bush 0.5% vs. Common milkweed 5%	< 0.0001
False indigo-bush 1% vs. Common milkweed 5%	0.0092
Common milkweed 5% vs. Common milkweed 0.5%	0.0044
**Lateral root density**	
False indigo-bush 0.5% vs. Common milkweed 5%	0.0033
